# Digitization and Linkage of PDF Formatted 12-Lead Electrocardiograms in Adult Congenital Heart Disease

**DOI:** 10.1016/j.cjcpc.2025.03.006

**Published:** 2025-04-01

**Authors:** Muhammet Alkan, Fani Deligianni, Christos Anagnostopoulos, Idris Zakariyya, Gruschen R. Veldtman

**Affiliations:** a School of Computing Science, University of Glasgow, Glasgow, United Kingdom; b Adult Congenital Heart Disease Clinic, Department of Cardiology, Helen DeVos Children’s' Hospital, Glasgow, United Kingdom; c Helen DeVos Children’s Hospital, Corewell Health, Grand Rapids, Michigan, USA

## Abstract

**Background:**

Twelve-lead electrocardiograms (ECGs) form an essential part of the late follow-up of patients with adult congenital heart disease (ACHD). Such ECGs are most frequently reviewed by clinicians in paper or PDF formats. These visual representations of the original vector data do not easily lend themselves to be directly analysed with the increasingly powerful machine learning algorithms that hold promise in risk prediction and early prevention of adverse events.

**Methods:**

In this work, we set out to create digital signals from ECG PDF documents by a series of data processing steps, validate accuracy of the process, and demonstrate its potential utility in research. Using 4153 ECG PDF documents from 436 patients with ACHD, we created a “pipeline” to successfully digitize the visually represented ECG vector datasets. We then proceeded with the validation of the digitized ECG dataset using several features that are also calculated by the vendor, such as QRS duration, PR interval, and ventricular rate, on all the patients.

**Results:**

We confirmed a strong correlation with the vendor measured ECG parameters including PR interval (R=0.941,P<0.05), QRS duration (R=0.949,P<0.05), and ventricular rate (R=0.971,P<0.05). Further, using support vector machine, a well-established machine learning model, we demonstrate the ability of the digitized ECG dataset to accurately predict anatomic diagnosis in ACHD.

**Conclusions:**

Digitization of PDF formatted ECG signal data can be accomplished with good accuracy and can be used in clinical research in ACHD.

## Résumé

**Contexte:**

Les électrocardiogrammes (ECG) à 12 dérivations font partie intégrante du suivi à long terme des patients atteints d’une cardiopathie congénitale chez l’adulte (CCA). Le plus souvent, les cliniciens interprètent ces ECG sur papier ou un document PDF. Ces représentations visuelles des données vectorielles d’origine ne se prêtent pas aisément à l’analyse directe au moyen d’algorithmes d’apprentissage machine toujours plus puissants, prometteurs pour la prédiction des risques et la prévention précoce des événements indésirables.

**Méthodologie:**

Dans le cadre de ce projet, nous avons entrepris de créer des signaux numériques à partir de rapports d’ECG au format PDF en suivant une série d’étapes de traitement des données. Nous avons également validé la précision du processus et démontré son utilité potentielle pour la recherche. Nous avons créé un pipeline de données à partir de 4 153 rapports d’ECG au format PDF provenant de 436 patients atteints d’une CCA et numérisé les ensembles de données vectorielles d’ECG représentés visuellement. Nous avons ensuite procédé à la validation de l’ensemble de données d’ECG numérisé à partir de plusieurs paramètres également calculés par le fournisseur, comme la durée du QRS, l’intervalle PR et la fréquence ventriculaire, pour tous les patients.

**Résultats:**

Nous avons été en mesure de confirmer une étroite corrélation avec les paramètres d’ECG mesurés par le fournisseur, soit l’intervalle PR (r=0,941;p<0,05), la durée du QRS (r=0,949;p<0,05) et la fréquence ventriculaire (r=0,971;p<0,05). En outre, à l’aide d’une machine à vecteurs de support, un modèle d’apprentissage machine bien établi, nous avons démontré que l’ensemble de données d’ECG numérisé permet de prédire avec précision le diagnostic anatomique de CCA.

**Conclusions:**

La numérisation des données de signal au format PDF peut être réalisée avec précision et utilisée dans la recherche clinique sur les CCA.

With advances in medical sciences, more than 90% of patients with even the most complex congenital heart disease (CHD) survive into adult life.^1^ With this transformational success of medical and surgical advances, a growing burden of morbidity and mortality has emerged as individuals enter later into adult life. Increasing attention is being drawn to the ability to predict which individuals are likely to deteriorate and which are at risk of mortality.^2^, ^3^, ^4^, ^5^, ^6^ Electrocardiograms (ECGs) are a fundamental aspect of such assessments.^7^, ^8^, ^9^, ^10^ They have been demonstrated to carry important diagnostic and prognostic information that correlate with clinically important outcomes.^10^, ^11^, ^12^, ^13^, ^14^ For example, in CHD, clinicians use derived ECG measures such as QRS duration, QRS fragmentation, QRS axis, and R-wave height along with PR intervals as indicators of underlying anatomy and prior surgery, disease state, and mortality prognosis in some specific settings.^15^

More recently machine learning (ML) algorithms have promised to extract relevant ECG features and exceed the performance of more traditional and more manual approaches in disease diagnosis and prognostication.^16^^,^^17^ In a recent review, Helman et al.^18^ highlighted that the development of ML algorithms to process 12-lead ECGs for CHD is a promising direction, which currently remains unexplored due to the lack of diverse and large datasets.

In our previous work, we have identified the opportunity to develop digital twins of patients with adult CHD by extracting information over a decade from clinical letters, which involved diagnoses, diagnostic complexity, interventions, arrhythmia, medications, and demographic data.^19^ We believe that this information can also be linked to the patient’s 12-lead ECG dataset when in digitized format. This provides the opportunity to develop robust ML models for diagnoses and risk prediction and explore their capabilities.

In clinical practice, 12-lead ECGs are often interpreted from scanned or printed PDF documents. Valuable information can be derived from such visual-manual assessment, such as cardiac rhythm and rate, haemodynamic characteristics like pulmonary hypertension, and prognostic information relating to sudden cardiac death risk. ML on the actual raw signal data enhances further diagnostic and prognostic capacity. Due to proprietary software and analysis of raw ECG signal data, such “raw” data are not readily available for *de novo* or empiric data analysis or use in further research. Being able to accurately reproduce the digitized ECG signals from PDF documents can potentially enable signal data to be used in developing ML models for risk stratification and diagnosis.

Once the ECG signal is acquired, it often contains noise and artefacts that need to be filtered out to obtain a clear signal.^20^^,^^21^ The most commonly used filters are low-pass, high-pass, and notch filters. Low-pass filters allow lower-frequency signals to pass through while blocking higher frequencies. They are typically set around 150 Hz, as the clinically relevant information in ECGs falls below this frequency.^20^ High-pass filters remove low-frequency noise, such as baseline wander, which can be caused by breathing or movement.^20^ Notch filters are used to remove specific frequency noise, such as the 50/60 Hz interference from power lines.^20^ After filtering, the analog ECG signal is converted into a digital format through a series of processes involving sampling the signal at regular intervals and quantizing the amplitude values to represent the signal digitally. The digitized ECG data can then be stored, analysed, and processed using various clinical tools and algorithms for further analysis and diagnosis.

For ECG digitization, all current approaches focus on image processing (ie, on scanned images or PDF documents converted into a desired image format, ie, processing the ECG as a “picture”), rather than the conversion to the vector data represented by the PDF document,^22^^,^^23^ and this in particular has not previously been evaluated in CHD.

We propose a stepwise approach as a pipeline (ie, workflow) that assembles several steps that are combined to extract 12-lead ECG digital signals from ECG PDF documents (see Central Illustration). We adopt open-source generic Python libraries to extract information about the text and the geometric objects present in ECG PDF documents, for example, PyMuPDF.^24^ We then validate the digitized dataset using derived ECG features such as QRS duration, PR interval, and heart rate, and further demonstrate its potential research utility by using it to predict anatomic diagnosis associated with CHD. This is an initial step towards outcome prediction, which requires significantly larger numbers of subjects to achieve.

## Methods

### Linkage of CHD EHR

**Figure 1:**
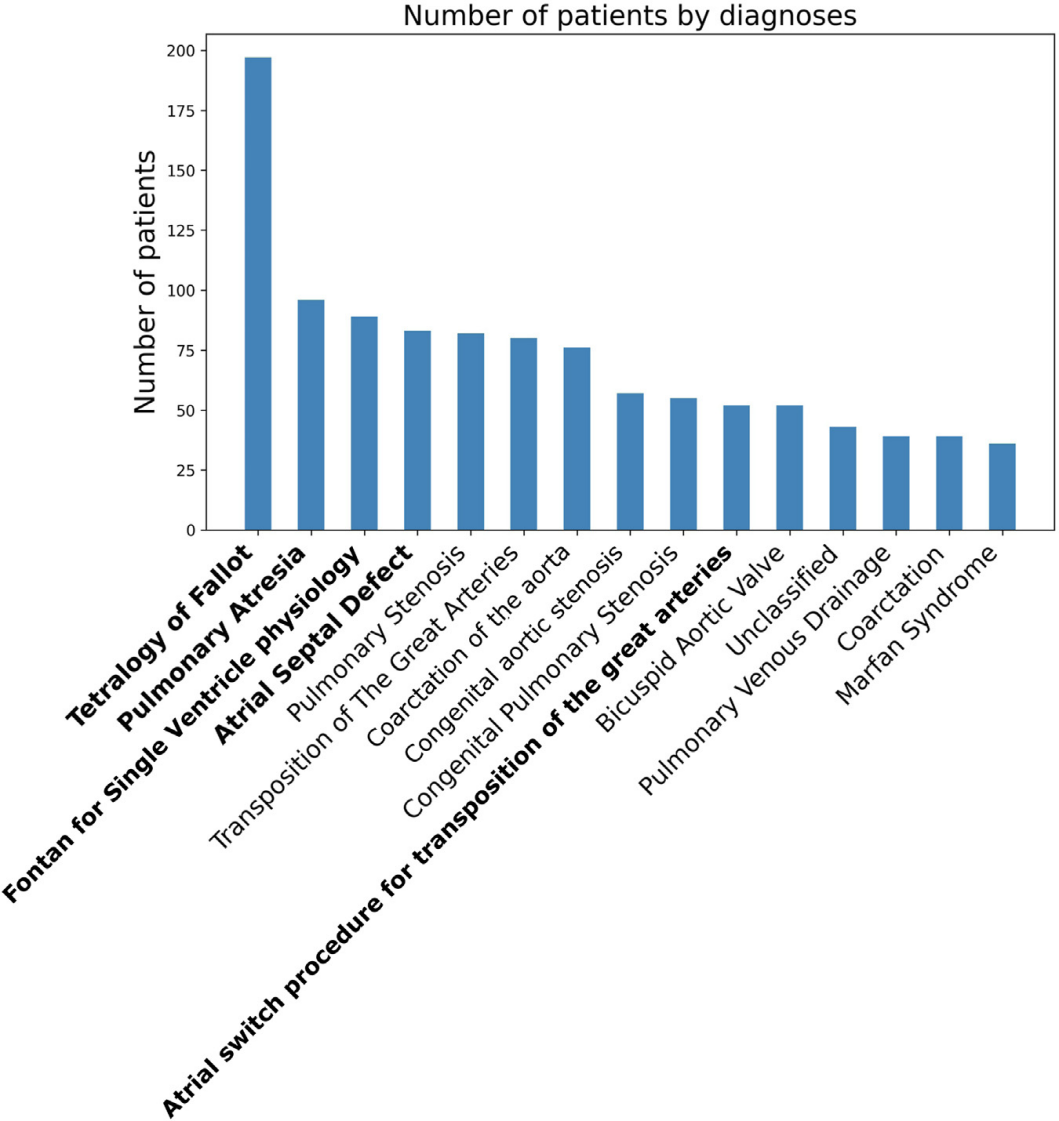
Top 15 anatomic CHD diagnoses for 1409 patients. Summary of all the patients initially screened for inclusion. CHD, congenital heart disease.

Study approval was obtained by the Institutional Governance Division of the NHS Golden Jubilee National Hospital. Demographic information including age, biological sex, anatomic diagnoses, and prior surgical intervention was extracted from clinical letters as previously described in the paper by Verma et al.^19^ The most common condition was tetralogy of Fallot (ToF) in 197 patients, followed by pulmonary atresia (PA) in 96 patients. The top 15 anatomic diagnoses are summarized in Figure 1.

Patients were selected based on the 5 most prevalent congenital heart diagnoses in our practice:

(1) ToF (diagnosis list)
(2) PA (diagnosis list)
(3) Fontan (intervention and diagnosis list)
(4) Atrial septal defect (ASD) (diagnosis list)
(5) Mustard (intervention list) including other atrial switch procedures.

Information extracted from outpatient clinic letters was linked to ECG PDFs based on the patient chart number. Patients were excluded if they did not have available ECGs or were in a permanent dysrhythmia, such as atrial fibrillation or flutter, or were atrioventricularly paced. This led to 436 patients being included in our investigation. Of these 436 patients, 173 had ToF, 77 had ASD, 73 had PA, 66 had Fontan, and 47 had Mustard. Overall, 4153 ECG PDF documents were extracted from these 436 patients with diagnoses as outlined.

### ECG data preprocessing

Twelve-lead ECG data were extracted from ECG PDF documents obtained via the Marquette 12SL by the GE Healthcare (Milwaukee, WI) analysis program.^25^ Standard 12-lead ECG placement was used, ensuring consistent and accurate readings across different patients. For resting ECGs, the analog voltage potential is digitized into 4.88 μV units at a rate of 4 kHz. The software downsamples the signal to 500 samples per second and represents a value every 0.05 mm on a chart. The ECG signal is then preprocessed to remove noise and QRS template matching used to extract ECG features and export ECG waveforms to PDF documents in a vectorized format. Each PDF document contains 12 leads in a specific order (leads I, II, III, aVF, aVR, aVL, V1, V2, V3, V4, V5, and V6) and provides only 2.5-second strip for each lead.

### ECG digitization process

**Figure 2:**
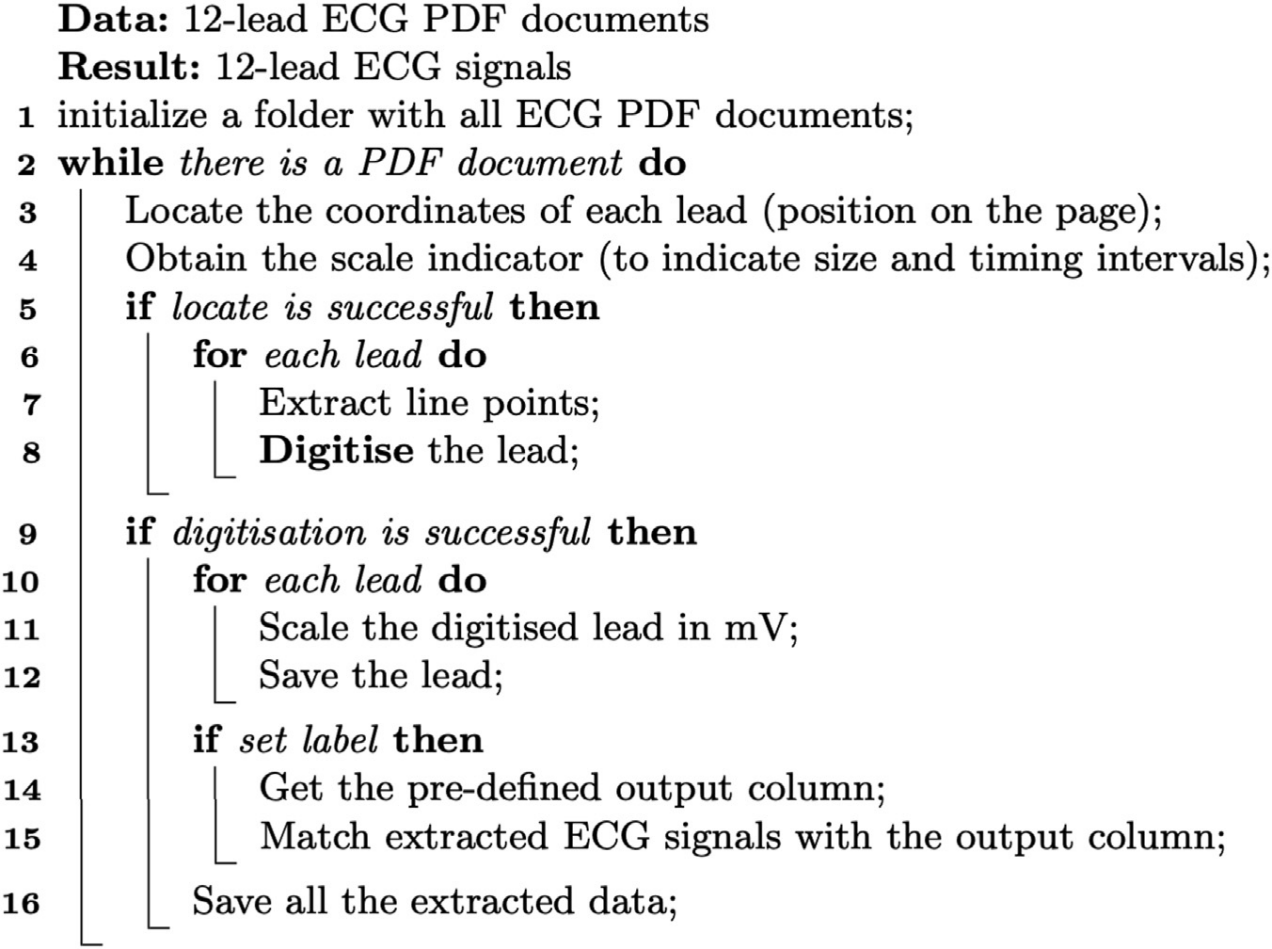
Algorithm 1: ECG digitization pseudocode. The column output was derived from linked outpatient clinic letters. ECG, electrocardiogram.

**Figure 3:**
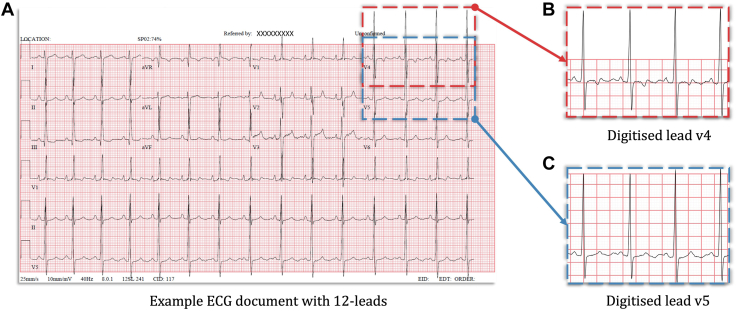
Digitization and linkage of ECG PDF documents. (**A**) A sample ECG PDF document that uses vectorized graphic format to store ECG waveforms. (**B,C**) The results of the digitized leads (for leads V4 and V5). ECG, electrocardiogram.

We developed a new algorithm using vector drawing software on the ECG PDF documents to digitize ECGs without user intervention. PyMuPDF^24^ library, another artificial intelligence software algorithm, was used to extract information about the graphics points present in all the PDF documents. The algorithm follows the steps detailed in Figure 2. An example ECG document with 2 of the extracted leads is shown in Figure 3.

### Digitized ECG analysis and ECG peak alignment

**Figure 4:**
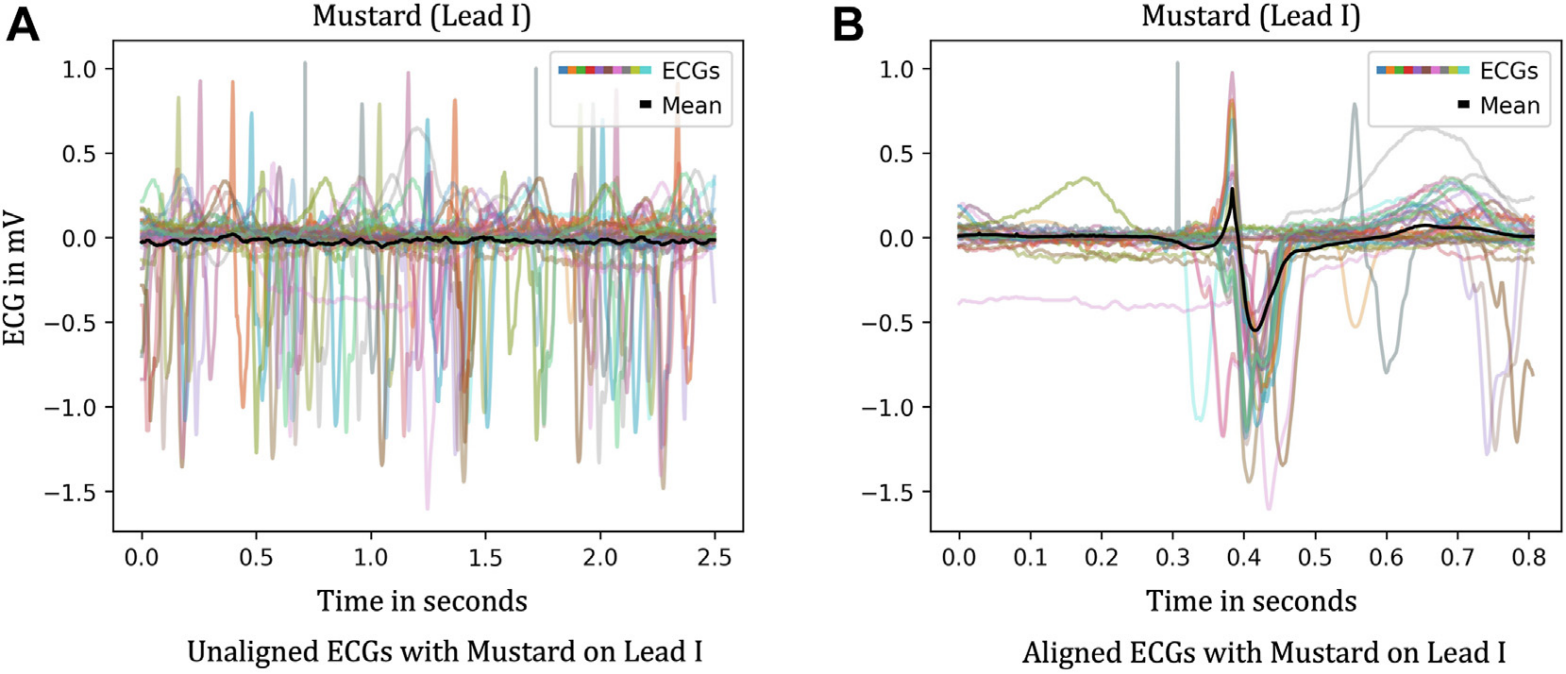
ECG alignment using random 50 ECGs on lead I of patients with a Mustard procedure for transposition of the great arteries. (**A**) Individual nonaligned ECGs for the random 50 patients. (**B**) QRS aligned ECGs with the overall signal vector (outlined in bold) for the same random 50 patients. ECG, electrocardiogram.

To facilitate analysis of ECG data with ML algorithms, we standardized them by *aligning* the ECGs so that the peaks of the R-waves intersected and QRS complexes were digitally synchronized across all leads. An example of the average signal of the aligned ECGs for Mustard is shown in Figure 4, as well as its characteristic of the underline abnormality.

For validation of our algorithm in Figure 2, we compared vendor-derived ECG intervals with derived intervals from the digitally aligned extracted ECG signals. All the patients were checked to assess the accuracy of the digitization algorithm.

**Figure 5:**
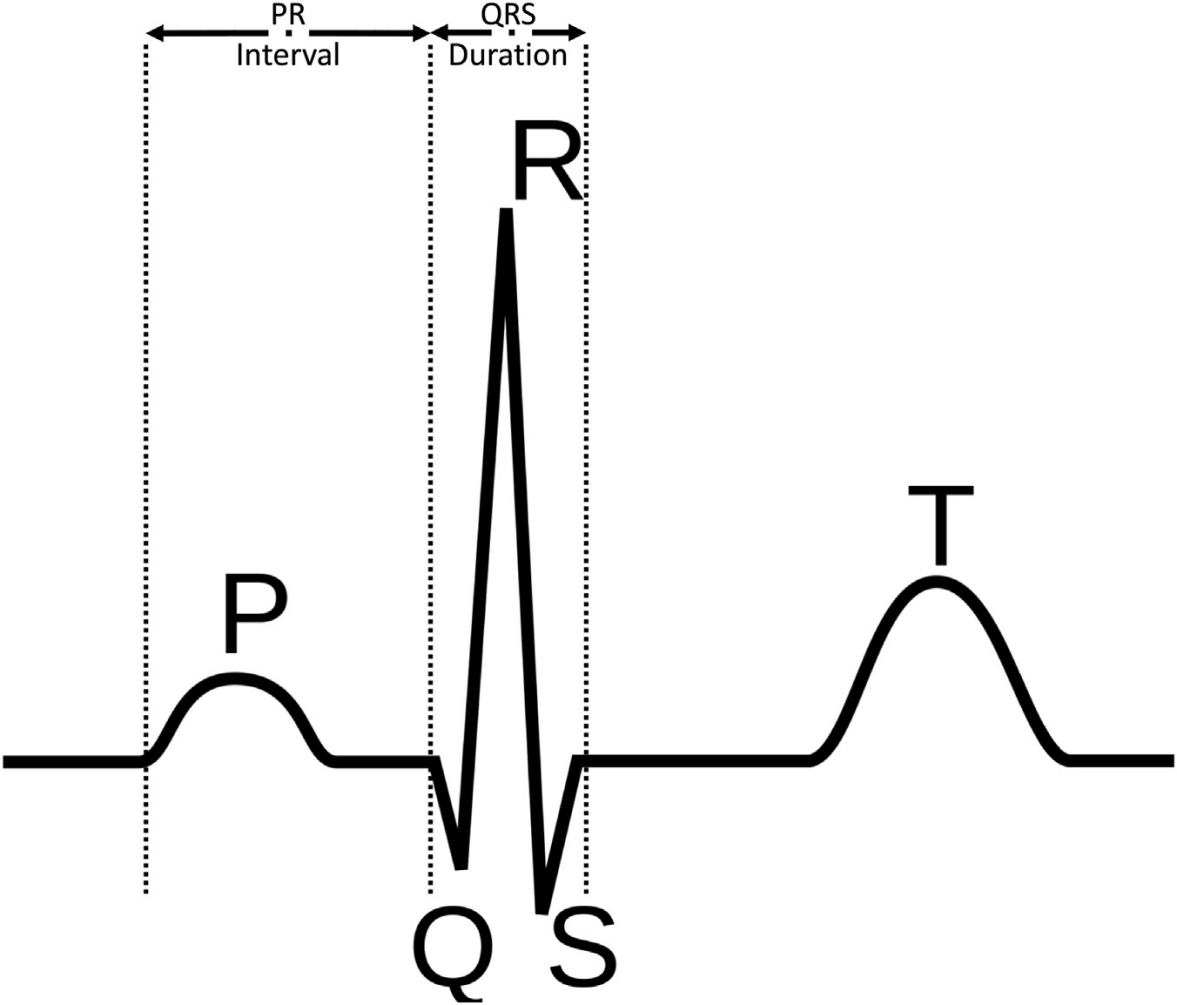
Demonstration of a normal single-lead ECG trace, with P, Q, R, S, and T points. ECG, electrocardiogram.

The onsets and offsets for the points P, Q, R, S, and T in all 12 leads should be determined first to enable us to calculate QRS duration, PR interval, and ventricular rate. An example illustration can be found in Figure 5 on a single beat. In the vendor algorithm,^25^ onsets are defined as the earliest deflection in any 12 leads, and offsets are defined as the latest deflection in any 12 leads. The QRS duration is measured in milliseconds from the earliest Q onset in any lead to the latest S offset in any lead. Similarly, the PR interval is measured in milliseconds from the earliest P onset in any lead to the QRS onset (or the earliest Q onset) in any lead. For the ventricular rate (beats per minute), the number of beats is counted and divided by the time difference in minutes between the first and last beat. For all our corresponding calculations of QRS duration, PR interval, and ventricular rate, the NeuroKit2^26^ library was used. We followed a processing pipeline similar to the vendor guidelines^25^ to enable meaningful comparison of the final measurements.

### Training to predict diagnosis

The support vector machine (SVM) model works by mapping training data into a high-dimensional space to maximize the gap between the points of different labels.^27^ The training of the SVM model was performed on the aligned ECG signals, as described above, to predict the corresponding diagnoses using the radial basis function kernel^28^ with regularization (C = 1).

**Figure 6:**
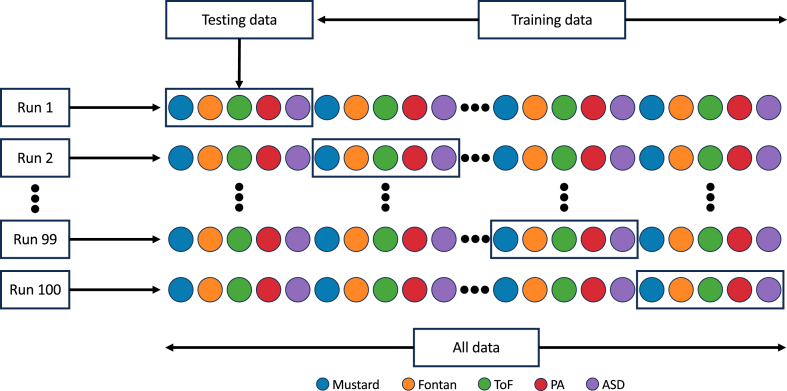
Stratified patient leave-out setup. Each colour represents a different diagnosis. Each **coloured circle** contains all the ECGs of a single patient with the corresponding diagnosis highlighted with the colour. Each run represents a training and testing cycle and the SVM model trained from scratch in each run. For each run, 5 patients are randomly selected, and all the data for these patients are kept for testing, whereas the rest are used for the training, from run 1 to 100. ASD, atrial septal defect; ECG, electrocardiogram; PA, pulmonary atresia; SVM, support vector machine; ToF, tetralogy of Fallot.

The training-testing split of data was repeated 100 times based on pseudorandomized, stratified patient leave-out evaluation to ensure that the testing set of patients was representative for all the classes. In other words, one patient from each class was randomly selected to populate the testing set, and all the remaining patients to populate the training set. For each run, the SVM model trained on the populated training set and then tested on the corresponding testing set that does not include any data from the patients in the training set (ie, stratified patient leave-out method). Using such an approach, summarized in Figure 6, stratified patient leave-out enhances the capability of the SVM model to predict unseen data by reducing bias.^29^ This approach ensures that each class is proportionally represented in both the training and test sets, thereby preventing overfitting and underfitting. A stratified patient leave-out approach was selected to facilitate the model’s ability to more effectively identify the characteristics associated with the disease itself, rather than those specific to the patients.

## Results

Of the 436 patients, 194 (44.4%) were female. The most common condition was ToF (39.9%), followed by ASD (17.6%) and PA with ventricular septal defect (16.7%). The mean age at ECG was 33 years (standard deviation: 11.7 years) and the 25%-75% interquartile range 23-40 years. In several ECGs, there were overlaps between individual lead signals on the original PDF documents, as can be seen in Figure 3. This included lead labels as well as large QRS complexes impinging on the lead displayed below it, in particular, the precordial leads (V2-V6). We developed an algorithm to successfully overcome this issue using the vector drawings on the original ECG PDF documents. Sample extraction results for leads V4 and V5 can be seen in Figure 3.

**Figure 7:**
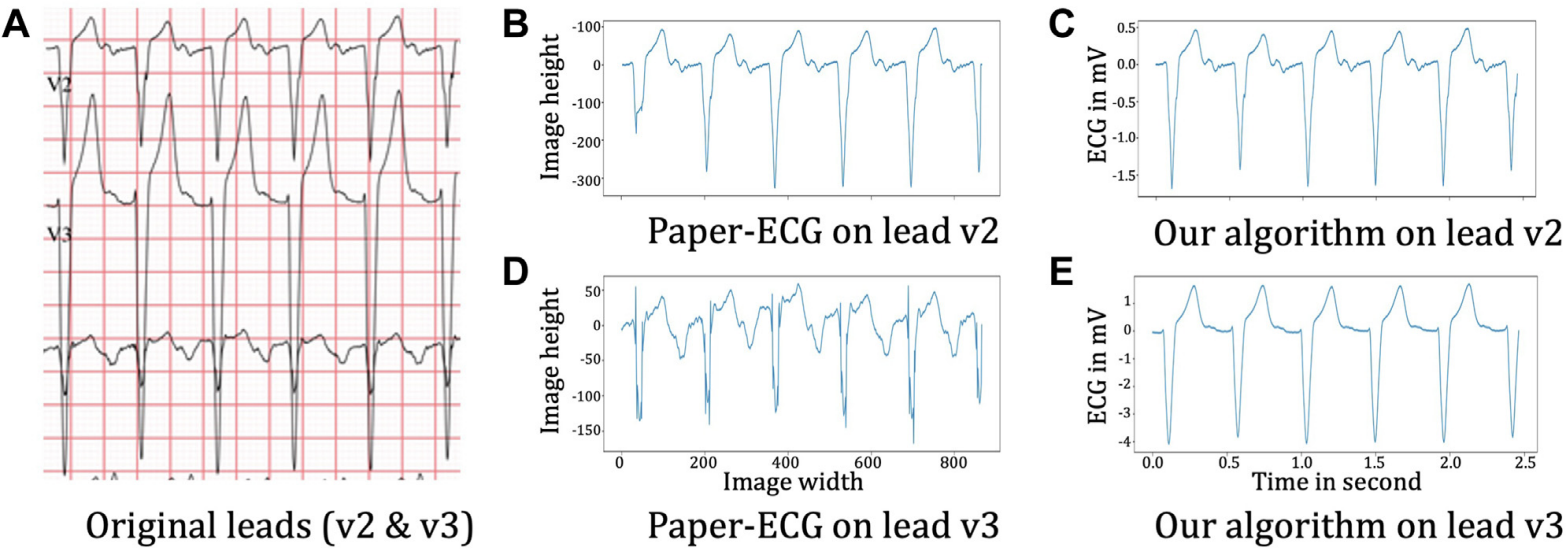
Comparison of our digitization results of the vectorized graphic-based ECGs. (**A**) Original signals from the corresponding ECG document for leads V2 and V3. (**B,D**) Digitization results of Paper-ECG.^30^ (**C,E**) Digitization results of our algorithm for the same leads V2 and V3, respectively. ECG, electrocardiogram.

Other artefacts were overcome without further user intervention, as shown in Figure 7 on another patient. We compared our work with prominent open-source approaches found in the literature to digitize ECGs like Paper-ECG.^30^ Figure 7 demonstrates the advantages of our methods by comparing ECG segment extraction of the 2 algorithms side-by-side, respectively. Original signals from the corresponding ECG documents are shown in Figure 7A for leads V2 and V3. All the original signals have some difficulties such as text overlaps of lead names and ECG notes, and signal overlaps between different leads. These difficulties may lead to the failure of digitization, as detailed in the following examples. Although the initial QRS complex in Figure 7A for V2 is distorted and abbreviated in a vertical direction in Figure 7B because of the lead name overlapping with the signal, our algorithm can correct this in Figure 7C. Figure 7D shows overlapping QRS complexes from the above lead impinging on the QRS complexes of the present lead. Our algorithm also corrects this in Figure 7E. Similarly, the baseline shift artefact in Figure 7D is corrected as shown in Figure 7E.

**Figure 8:**
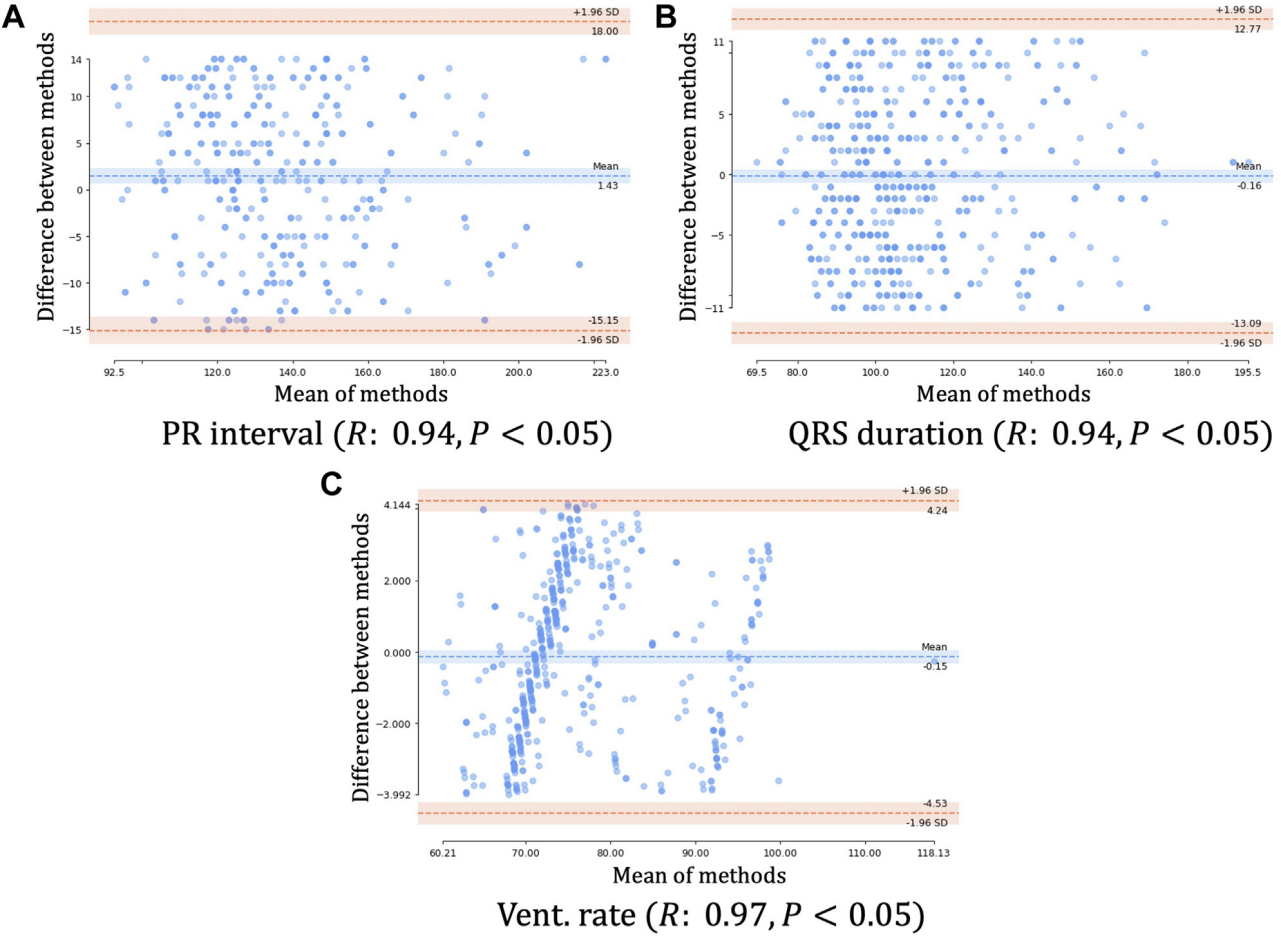
Bland-Altman plots between vendor and extracted values. PR interval, QRS duration, and ventricular rate are calculated, and Bland-Altman plots are plotted in (**A**), (**B**), and (**C**), respectively. Plots depict the agreement between the vendor calculated values on original signals and user calculated values on extracted signals. It also shows that there is no bias as the mean difference between 2 measurements is not consistently positive or negative.

Bland-Altman plots are plotted to compare the measurement of the 3 variables using 2 different algorithms, vendor and our implementation. The mean of the 2 measurements is plotted on the *x*-coordinate, whereas the *y*-axis is the difference between the 2 algorithms, to show the agreement or disagreement between the 2 results. PR interval, QRS duration and ventricular rate are calculated and plotted in Figure 8A-C, respectively. For all the calculations, similar techniques as in the vendor manual^25^ were implemented, as closely as possible. Ventricular rates derived from the digitized ECG correlated well with the original vendor-rendered ECG, as can be seen in Figure 8A. Similarly, in Figure 8B and C, the correlation between vendor calculated and digitized ECG calculated values for both QRS duration and PR interval is displayed, respectively. Plots depict the agreement between the vendor calculated values on original signals and the user calculated values on extracted signals. They also show that there is no bias as the mean difference between 2 measurements is not consistently positive or negative. Pearson correlation coefficients are also reported along with the 2-sided *P* value, to show that there is a strong correlation between the results. We also performed a null hypothesis test to calculate the significance of the correlation coefficient and to decide whether the relationship between the results is strong enough to be used to model the relationship. Null hypothesis assumes that the correlation coefficient is not significantly different from zero, and hence there is no significant relationship between the variables. As the *P* value is less than the chosen significance level (α = 0.05), we reject the null hypothesis. It indicates that there is sufficient evidence to conclude that there is a statistically significant correlation between the 2 results.

Pre- and post-R-peak alignment differences are shown in Figure 4, along with the mean indicated by the black line. Improvements in consistency of ML performance were observed with the help of alignment on R-peaks, and the aligned data were much more successful in classification results.

**Figure 9:**
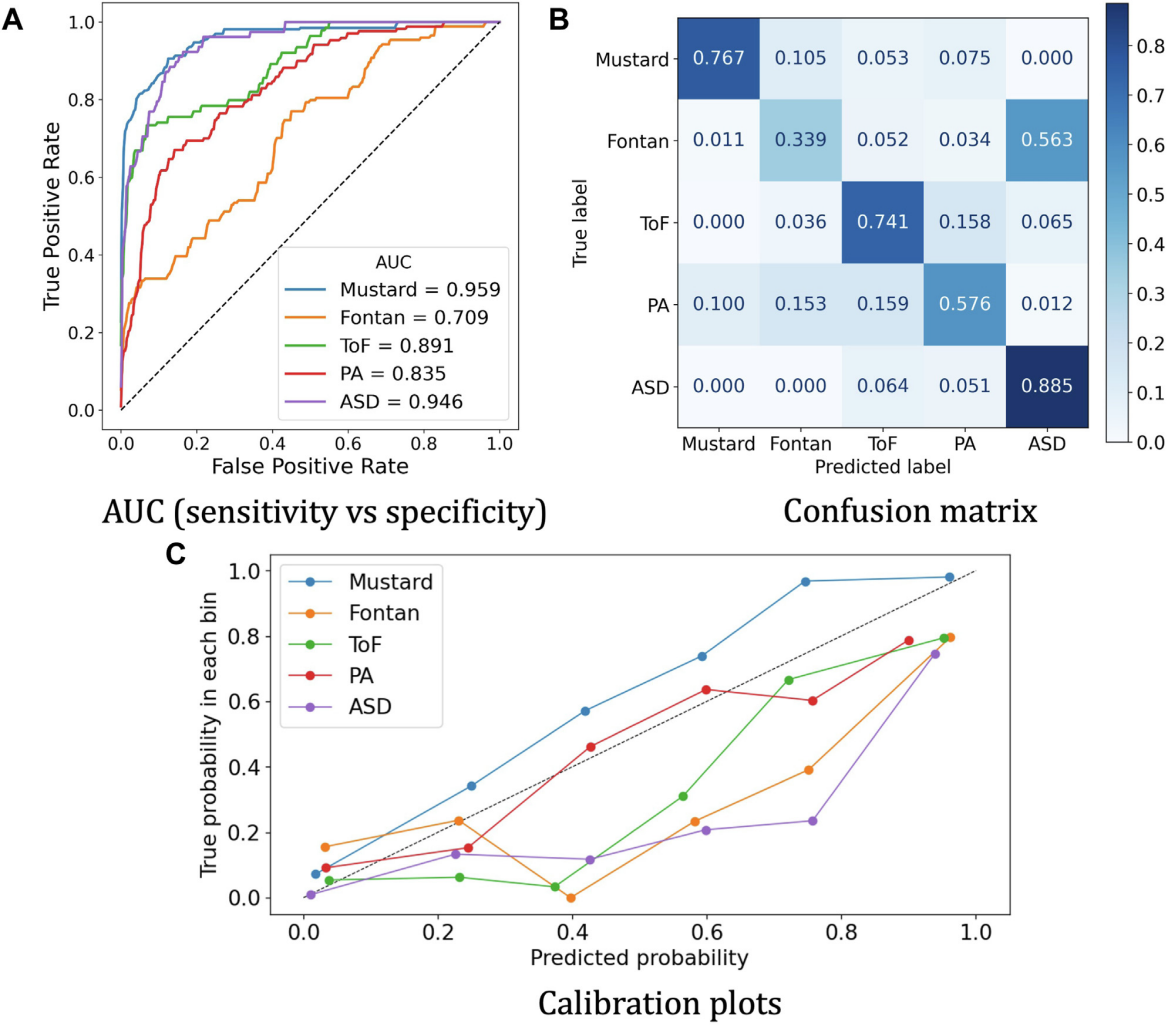
Classification performance of the SVM model. (**A**) The average area under the curve (AUC) scores of all 100 runs for each class are displayed separately. (**B**) Confusion matrix of all 100 runs combined is displayed to highlight model’s prediction vs true label. (**C**) Calibration plots for each class are displayed separately. ASD, atrial septal defect; PA, pulmonary atresia; ToF, tetralogy of Fallot.

The ability of the model to predict diagnosis accurately is displayed in Figure 9. Figure 9A shows the performance of the classification on the area under the curve (AUC) plot. It is a metric for assessing a binary classifier’s capacity to differentiate between classes by demonstrating the true-positive against the false-positive rate. As AUC scores are typically used in binary classification, the one-vs-rest strategy is used to evaluate AUC scores for each class separately. For example, the diagonal dashed line depicts the AUC curve of a random predictor that has a score of 0.5.

**Central Illustration:**
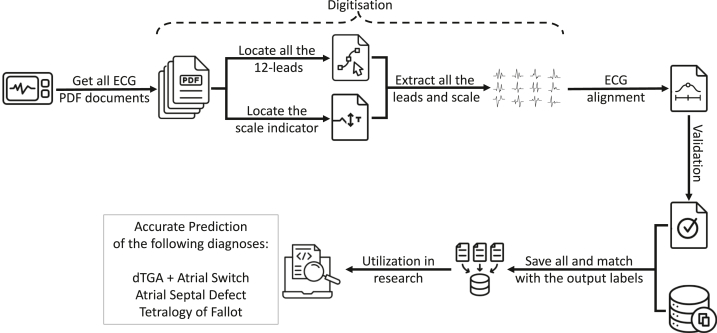
Overall workflow from digitization to utilization in research. Selected set of ECG PDF documents are placed in an analysis file. Then, digitization is applied to extract all the leads and all the extracted data is stored for later use. Alignment and validation steps are also performed to ensure that there are no problems, and then all the data is matched with the corresponding output labels. After all these steps, it is now fully digitized and stored in a format that can be utilised in research.

Moreover, the confusion matrix for the SVM model can be seen in Figure 9B. The confusion matrix helps to understand the classes that are misclassified by presenting the model’s prediction summary in matrix form, showing how many predictions per class are correct and how many are incorrect. The model predicted a diagnosis of ASD accurately in 88% of cases, dextro-transposition of the great arteries with a Mustard operation in 76%, ToF in 74%, PA in 57%, whereas in single ventricle circulations with a Fontan circulation only 34% of cases. All the classification metrics, including precision, sensitivity, specificity, and F1 score, are reported in Table 1.

**Table 1:** Classification metrics for all the classes

	Precision	Sensitivity	Specificity	F1 score
Mustard	0.915	0.966	0.767	0.834
Fontan	0.5	0.909	0.339	0.404
ToF	0.652	0.92	0.741	0.694
PA	0.653	0.92	0.576	0.612
ASD	0.388	0.854	0.885	0.539

ASD, atrial septal defect; PA, pulmonary atresia; ToF, tetralogy of Fallot.

## Discussion

ECGs are powerful tools when used at a population scale to identify poor cardiovascular outcomes. ECGs can be used to provide a sense of the physiological and structural condition of the heart, while also providing valuable diagnostic clues. Currently, analysis of large-scale data requires access to the original ECG datasets, which is embedded in a codified fashion within the vendor analysis software and is not readily accessible for analysis. Instead, clinicians tend to use manual evaluation of printed ECGs or review of ECG PDF documents for interpretation. Digitization of such 12-lead ECGs, as we have been able to demonstrate in this article, is potentially very helpful in facilitating larger scale research including risk stratification, cross-institutional research, and registry data recording.

There are only a few studies on CHD (for adults or children) that focus on ECG analysis and classification using ML techniques due to the lack of diverse and large labelled data. Most previous works were implemented as a binary problem to detect whether a patient has the condition or not. For example, Du et al.^16^ proposed to use ECG for CHD screening in children. On the other hand, Khan et al.^17^ proposed to identify common congenital heart defects and distinguished them from normal ECGs. The authors proposed to extract ECG features such as mean, root mean square, peak-to-peak, and signal-to-noise ratio for prediction. Finally, Diller et al.^10^ proposed a deep learning architecture to categorize the diagnostic group, disease complexity, and New York Heart Association class in a large cohort of patients with adult CHD only including some ECG parameters such as resting heart rate, QRS duration, and QTc duration along with laboratory and exercise parameters.

To our knowledge, this is the first time that ECG data from patients with CHD are extracted from PDF documents and labelled “automatically.” Our proposed framework does not require additional user manipulation, and we have extensively validated it by estimating ECG features, such as PR interval, QRS duration, and ventricular rate, and comparing the values with the vendor corresponding values.

There are several digitization algorithms implemented by different researchers and vendors,^22^^,^^23^^,^^30^ but, to our knowledge, all of them work on pixelated images captured from the ECG PDF documents, or from the scanned ECG papers. This often results in loss of quality of the original ECG image digitized signal due to a variety of factors such as interpretive text that is displayed alongside the ECG overlapping with the ECG thereby diminishing accurate digitization. Here we used open-source tools to extract ECG vectorized graphical information from the PDF documents. In this way, we were able to reconstruct the signal accurately.

The digitized ECG overcomes artefacts present in the Paper-ECG^30^ digitized ECG traces in Figure 7 and accurately corrects these without any user intervention. For the first time, we demonstrate the ability of a relatively simple ML model to reasonably predict diagnosis in the selected 5 conditions including ASD, Mustard, single ventricle physiology with a Fontan circulation, PA, and ToF. This preliminary investigative application and utility demonstrates encouraging potential for the digitized ECGs to be useful in the research setting. We believe that digitization of the ECG will facilitate further research used in large datasets that can use PDF formats of the ECG.

The ability of the algorithm we developed to correct artefacts on the original ECG document was particularly gratifying. We demonstrate the ability to correct for overlapping QRS complexes, baseline drift in the ECG, and text overlapping QRS or other parts of the ECG traces in patients with CHD. Although there are open-source works in the literature to digitize ECGs like Paper-ECG,^30^ all such software currently needs user intervention to locate all the 12-leads with a corresponding bounding box. With our automated approach, we can not only digitize but also export the corrected ECG waveforms to PDF documents to avoid overlapping and allow clearer interpretation. So, it allows us to reconfigure the 12 ECG leads from the PDF raw data including individual lead vector data rather than having just an image for each lead as we are able to capture all the vectors on the PDF document.

We validated the ECG digitization process by comparing vendor-rendered ECG data such as QRS duration, ventricular rate, and PR interval between the original ECG and the digitized version. We were able to demonstrate a strong correlation between the measurements. Inherent variability in the intervals is expected as the techniques used by the vendor to measure particular intervals vary considerably. For example, some vendors use only simple band-pass filtering, whereas others use template matching to detect QRS complexes.

In our case, raw ECG data were not readily available even for data analysis purposes. This is the main reason why we implemented such a digitization pipeline to enable further research on the raw data. We are also aware that this is not always the case, depending on the agreement with the vendor. Furthermore, this is a common practice among wearable technology vendors, who typically provide only PDF reports rather than raw data. By developing capabilities to extract structured data from these PDFs, interoperability can be enhanced across different systems and platforms. Our digitization pipeline can be easily updated for different PDF structures/layouts of ECGs. We have tried several open-source works and noncommercial toolboxes to digitize ECGs (like Paper-ECG^30^), but the results were poor on the patients with CHD. The ML model was a way to prove the algorithm works, and it was sufficient for a smaller set of patients, more like a proof of concept.

We were able to use ML to train a relatively simple SVM model to recognize underlying cardiac anatomy such as in ToF, transposition of the great arteries with an atrial switch procedure, and, to a lesser extent, patients with single ventricle physiology, and a Fontan procedure where the discriminative ability was poor. Perhaps this is not unexpected given the enormous variety of underlying anatomic variation seen in patients with single ventricle physiology. We also had relatively few ECG data to analyse for individual lesions, and this could be greatly enhanced with larger scale datasets, which is the next step in this research process.

### Limitations

The process currently used to analyse ECG documents still requires manual deposition of the relevant 12-lead ECG documents into an analysis folder. Furthermore, despite the encouraging findings of this research, our total number of patients was still relatively small, hindering more robust training of the ML algorithm. Automation of this process would improve efficiency. Another limitation that should be addressed is the data extracted for this study, as the data used comprise average vector and time interval data rather than the real raw signals. Finally, our proposed method has been developed on PDF documents that encode the ECG waveforms in a vectorized format. Therefore, our method cannot improve the extraction of ECG waveforms when the ECGs have been digitized as pixelated images.

## Conclusions

We have adapted an extraction algorithm to digitize ECG PDF documents accurately without any user intervention. Our digitization algorithm not only extracts ECG leads accurately but also provides further flexibility, allowing the ECG PDF document to be reconstructed as requested. Also, using a rather simple ML algorithm, we demonstrated promising results on 12-lead ECG classification of anatomic diagnosis in CHD. Classification yields better results in terms of class distinction/discrimination on the aligned ECG signals, and it will be further tested on deep learning models to improve classification performance. Next steps would be to use this approach for risk stratification of mortality/cardiovascular hospitalizations and cardiovascular worsening in larger patient cohorts.
